# Underwater resection of cecal submucosal tumors using cap, clip, and snare assistance

**DOI:** 10.1055/a-2584-1496

**Published:** 2025-05-22

**Authors:** Chang-en Liu, Yan Li

**Affiliations:** 1 74672 Department of Gastroenterology and Hepatology, The Third Central Hospital of Tianjin, Tianjin, China

Clip-and-snare assisted endoscopic mucosal resection (CS-EMR) is a safe and effective treatment for small rectal neuroendocrine tumors (NETs) 1 . Following this approach, we applied the underwater method, U-CCS-EMR, for resecting submucosal lesions in the cecum.

A 42-year-old asymptomatic woman was identified with a 5 mm yellow, elevated submucosal lesion situated at the cecum during a routine colonoscopy. Ultrasonography (12 MHz) confirmed the lesionʼs location within the submucosal layer ( Fig. 1 ). Endoscopic resection was requested.

**Fig. 1:**
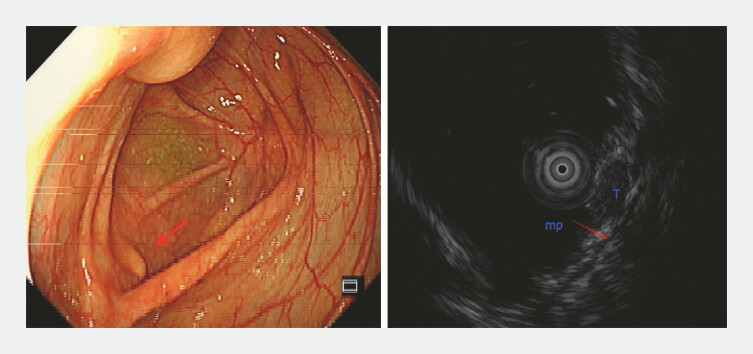
Yellow submucosal lesion (red arrow) confirmed by ultrasound to be located in the submucosal layer.

A transparent cap-covered single-channel colonoscopy, along with a pre-anchored snare, was inserted into the ileocecal region to target the lesion. The lesion, in a low-lying position, was obscured by residual fecal water, which significantly hindered both observation and treatment. Consequently, we opted for an innovative underwater treatment method ( Video 1 ). A clip was introduced via the working channel of the endoscope to secure the mucosa adjacent to the lesion ( Fig. 2 ). When the lesion and surrounding tissues were well lifted by the clip, the snare was released from the transparent cap and completely enveloped the root of the lesion ( Fig. 3 ). The lesion was entirely resected en bloc, leaving a clean surgical wound. The wound was promptly closed using clips. The patient was placed on a 24-hour fast following the procedure and was discharged in good condition 2 days later with no complications.

Endoscopic removal of the cecal submucosal tumor.Video 1

**Fig. 2:**
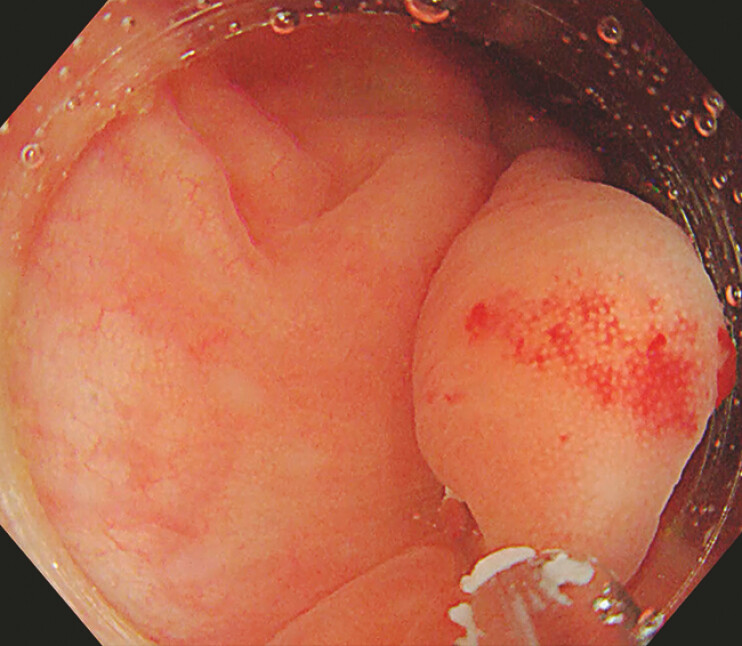
A clip was placed via the endoscope to secure the mucosa under the lesion.

**Fig. 3:**
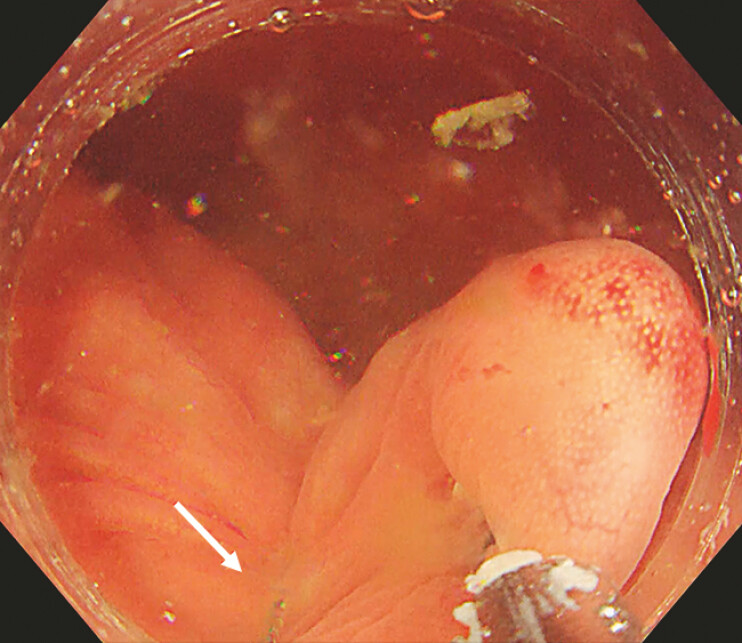
Release the snare and position it at the base of the pseudo-pedicle.

U-CCS-EMR is a promising treatment for cecal submucosal tumors. It provides a clear view throughout the procedure, especially during bleeding ( Fig. 4 ). It also preserves the submucosal layer’s natural laxity, aiding in the formation of a pseudo-long pedicle and ensuring a negative resection margin. Postoperative pathology revealed the lesion to be a granular cell tumor ( Fig. 5 ). Additionally, it reduces thermal damage to the muscle layer, lowering the risk of delayed perforation. Our experience shows that underwater resection with cap, clip, and snare assistance is both safe and effective.

**Fig. 4:**
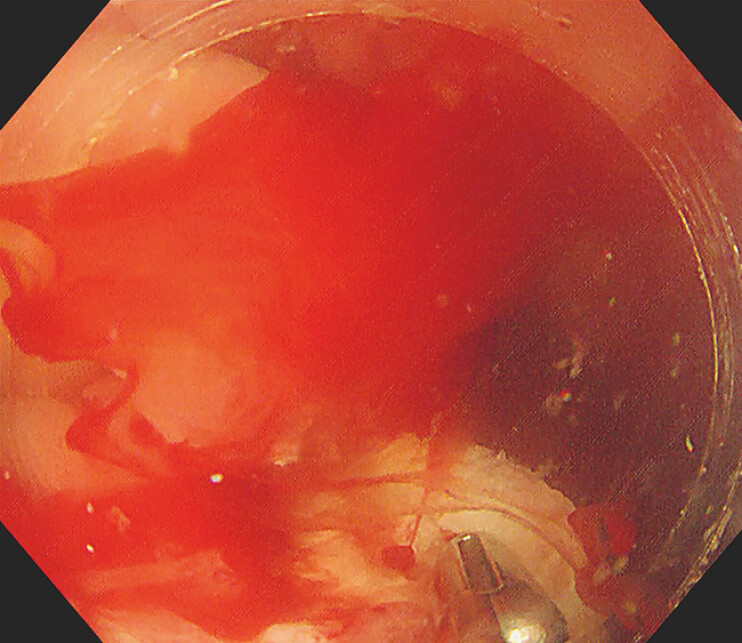
Underwater view: clearly visible low-position lesion and mini arterial bleeding point.

**Fig. 5:**
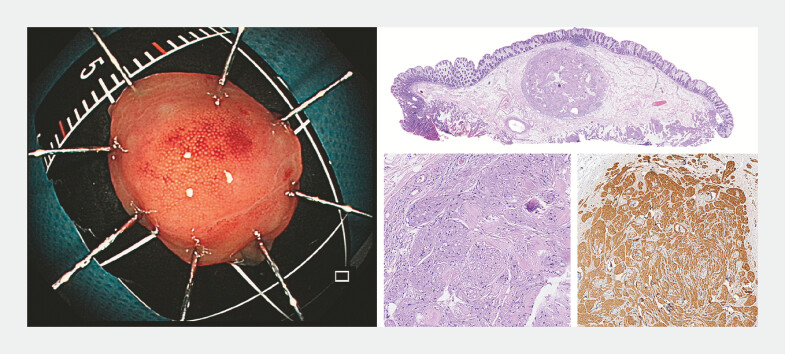
The lesion was resected en bloc and histopathology indicated granular cell tumor: HE, HE (20×), and S100 (+).

Endoscopy_UCTN_Code_TTT_1AQ_2AD_3AF

## Notes

Endoscopy E-Videos https://eref.thieme.de/e-videos
*E-Videos* is an open access online section of the journal *Endoscopy* , reporting on interesting cases and new techniques in gastroenterological endoscopy. All papers include a high-quality video and are published with a Creative Commons CC-BY license. Endoscopy E-Videos qualify for HINARI discounts and waivers and eligibility is automatically checked during the submission process. We grant 100% waivers to articles whose corresponding authors are based in Group A countries and 50% waivers to those who are based in Group B countries as classified by Research4Life (see: https://www.research4life.org/access/eligibility/ ). This section has its own submission website at https://mc.manuscriptcentral.com/e-videos .
